# Long non-coding RNA U90926 modulates IFN-γ-stimulated gene transcription and cell-intrinsic anti-*Cryptosporidium* defense in intestinal epithelial cells

**DOI:** 10.1128/iai.00328-25

**Published:** 2025-09-22

**Authors:** Marion L. Graham, Ai-Yu Gong, Kehua Jin, Chansorena Pok, Zinat Sharmin, Juliane K. Strauss-Soukup, Xian-Ming Chen

**Affiliations:** 1 Department of Microbial Pathogens and Immunity, Rush University Medical Center 2468, Chicago, Illinois, USA; 2 Department of Biochemistry and Molecular Biology, School of Basic Medical Sciences, Hubei University of Science and Technology 418442, Xianning, Hubei, China; 3 College of Dental Medicine-Illinois, Midwestern University 69281, Downers Grove, Illinois, USA; 4 Department of Chemistry and Biochemistry, Creighton University College of Arts and Sciences 357852, Omaha, Nebraska, USA; University of California Merced, Merced, California, USA

**Keywords:** lncRNA, innate defense, *Cryptosporidium*, intestinal epithelium, U90926, IFN-γ

## Abstract

*Cryptosporidium* infects the intestine in a wide variety of vertebrates, and intestinal epithelial cells provide the first line of defense against *Cryptosporidium* infection. Interferon gamma (IFN-γ) from immune cells infiltrated at the site of infection plays a key role in the epithelial cell-intrinsic defense. Nevertheless, the success of the parasite is the result of its ability to evade the host immune responses. Increasing evidence suggests that long noncoding RNAs (lncRNA) participate in host-pathogen interactions, but the underlying mechanisms are not fully understood. We previously demonstrated that lncRNA U90926 is upregulated in response to infection but appears to be playing a pro-parasitic role given its ability to repress transcription of defense genes and aid the parasite during infection. We show here that inhibition of U90926 during *Cryptosporidium* infection increased expressions of *Irgm2*, *Igtp*, and *Iigp1*, which are known IFN-γ-stimulated genes, in a gene-specific manner. Depletion of U90926 results in an increase in histone modifications associated with gene transactivation in the promoter regions of *Irgm2*, *Igtp*, and *Ilgp1*, suggesting U90926 is regulating defense gene expression via epigenetic modifications. U90926 can interact with Ehmt2, a potent euchromatic methyltransferase, in the promoter region of these defense genes to alter histone modifications. Knockout of U90926 enhances IFN-γ-mediated inhibition of *Cryptosporidium* infection, suggesting that U90926 may modulate IFN-γ-induced gene expression to suppress cell-intrinsic antimicrobial defenses. The data highlight a strategy *Cryptosporidium* has evolved to hijack host cell lncRNA machinery to suppress the immune response and allow for a robust infection.

## INTRODUCTION

*Cryptosporidium* is a protozoan parasite that infects mammalian gastrointestinal epithelium and other mucosal surfaces (1). It is one of the leading pathogens responsible for moderate-to-severe diarrhea in children, particularly under the age of 2, and can predispose them to long-lasting defects in body growth and cognitive ability (2–5). While *Cryptosporidium* infection in immunocompetent adults is self-limiting, it is detrimental to AIDS patients and others with compromised immune systems (6). There is currently no fully effective treatment or vaccine, and waterborne transmission grants the parasite the potential to cause large-scale outbreaks and epidemics in both developed and developing countries (7–9). The majority of human *Cryptosporidium* infections are caused by two species: *C. parvum* and *C. hominis* (6, 10). *Cryptosporidium* oocysts are ingested from contaminated water or food and excysted in the gastrointestinal tract to release infective sporozoites. The sporozoites attach to the apical membrane of the intestinal epithelial cells and form an intracellular but extra-cytoplasmic parasitophorous vacuole where the organisms remain and further develop (10, 11). Therefore, it is the intestinal epithelial cells that provide the first line of defense and play a key role in initiating and regulating both the innate and adaptive anti-*Cryptosporidium* defenses (12).

 Gastrointestinal epithelial cells along the mucosal surfaces are integral components of cell-intrinsic immunity and a highly regulated communication network involving essential signals to and from the underlying cells of the gastrointestinal mucosa (13). Upon microbial challenge, the activation of pattern recognition receptors in intestinal epithelial cells results in a series of immune reactions, including the release of various cytokines/chemokines and the production of anti-microbial factors (14). The interferon (IFN) family of cytokines has been shown to play a critical role in the anti-*Cryptosporidium* defense. Interferons can be categorized into three categories: type I (IFN-α and IFN-β), type II (IFN-γ), and type III (IFN-λ family) (15). The activation of various types of IFN signaling pathways is not mutually exclusive, but evidence suggests that IFN-γ plays a crucial role in anti-bacterial and -parasitic immunity, whereas type I and III IFNs are primarily associated with anti-viral immunity (15–18). IFN-γ is produced by immune cells infiltrated at the site of infection, primarily type I innate lymphoid cells and T cells, and binds to the heterodimeric receptor of IFNGR1 and IFNGR2 expressed on intestinal epithelial cells (19–21). Canonical IFN-γ signaling employs the JAK/STAT pathway, leading to the activation of STAT1 and the subsequent formation of active STAT1 homodimers. The STAT1 homodimers then bind to the gamma-activated sites (GASs) located in the promoters or enhancers of IFN-γ-stimulated genes (ISGs) (18, 22). ISGs have a broad spectrum of cell-intrinsic responses aimed at combating intracellular pathogens including the production of potent defense molecules like reactive oxygen and nitrogen species, immunity-related GTPases (IRGs), and guanylate-binding proteins (GBPs) (16, 17, 23–25). Nevertheless, the success of the parasite is the result of its ability to evade these host immune responses while still utilizing host resources. The host and the parasite partake in a co-evolutionary “arms race” in which the parasite evolves novel strategies to escape the immune response, and the immune system simultaneously evolves strategies to kill the parasite. This dynamic competition results in the parasite employing innovative ways of utilizing host resources and machinery. Indeed, to counteract IFN-γ-mediated defense responses, *Cryptosporidium* has evolved strategies to dysregulate IFN-γ signaling in infected intestinal epithelial cells (26).

 Long noncoding RNAs (lncRNAs), transcripts over 200 nucleotides in length that are not transcribed into proteins, have been found to play a functional role in many different eukaryotic cells, including intestinal epithelial cells (27, 28). Increasing evidence suggests that lncRNAs can function to regulate gene transcription through specific interactions with cellular factors, including DNA, other RNA molecules, and RNA-binding proteins (RBPs) via nucleotide base pairing or the formation of structural domains generated by RNA folding (29–31). Several lncRNAs have been shown to play a role in regulating the innate immune response and can be targets of inflammatory pathways, thus altering their expression during microbial infection (32, 33). Our lab previously identified a host lncRNA, U90926, which was upregulated during *C. parvum* infection and appears to be playing a pro-parasitic role during *C. parvum* infection. U90926 was found to regulate expression of some host defense genes through histone modifications at the promoter region of the genes. Among those defense genes regulated by U90926 was adipocyte enhancer-binding protein 1, a modulator of NF-κB signaling and inflammation not previously shown to be involved in the anti-*Cryptosporidium* defense (34). However, the role of U90926 in IFN-γ mediated defense is unknown.

In this study, we sought to determine if U90926 is playing a role in IFN-γ-stimulated gene expression and identify the underlying mechanisms of regulation. Our findings indicate that U90926 is targeting certain ISGs to decrease their gene expression levels during *C. parvum* infection in a gene-specific manner and not by targeting the entire JAK/STAT signaling pathway. U90926 appears to be localizing to the promoter region of some ISGs to alter histone modifications associated with gene transactivation. U90926 may interact with Ehmt2, a euchromatic histone-lysine N-methyltransferase, increasing its enrichment in the promoter region of some ISGs to ultimately inhibit the IFN-γ-mediated intestinal epithelial cell-intrinsic anti-*Cryptosporidium* defense.

## RESULTS

### U90926 knockout alters gene expression profiles in intestinal epithelial cells in response to IFN-γ stimulation

**Fig 1:**
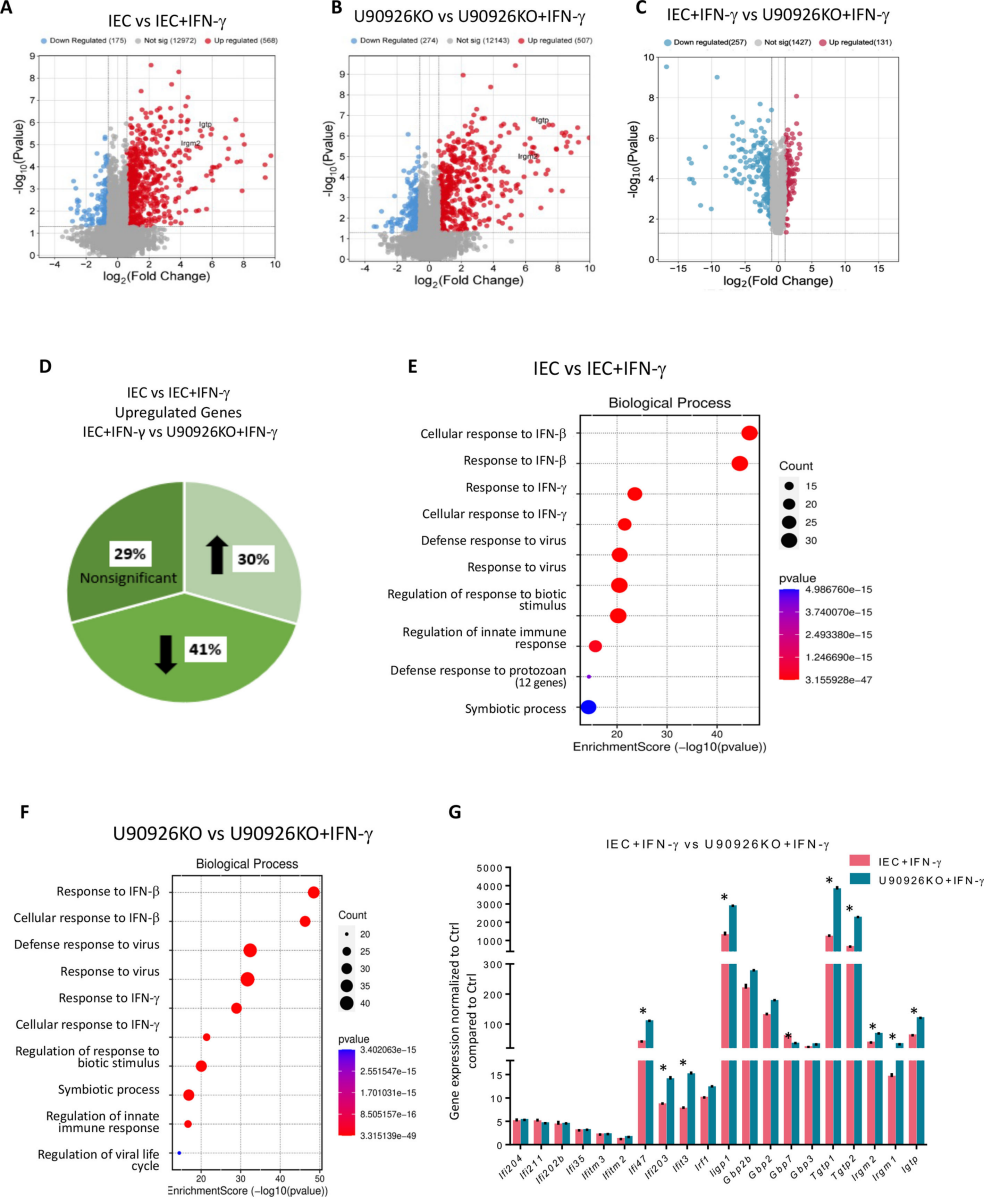
U90926 knockout alters the gene expression profiles in intestinal epithelial cells following the IFN-γ stimulation. IEC4.1 control and U90926KO cells were treated with IFN-γ (1 ng/mL for 4 h), followed by RNA-sequencing analysis. (**A**) Volcano plot of significantly altered genes in IEC4.1 IFN-γ-stimulated samples compared to non-treated samples (adjusted *P* < 0.05 with a fold-change > 1.5). (**B**) Volcano plot of significantly altered genes in U90926KO IFN-γ-stimulated samples compared to non-treated samples (adjusted *P* < 0.05 with a fold change > 1.5). (**C**) Volcano plot of significantly altered genes in U90926KO IFN-γ-stimulated samples compared to IEC4.1 IFN-γ-stimulated samples (adjusted *P* < 0.05 with a fold-change > 1.5). (**D**) Percentage of alterations in U90926KO IFN-γ-stimulated samples compared to U90926 non-treated samples of significantly upregulated genes in IEC4.1 IFN-γ-stimulated samples compared to IEC4.1 control cells. (**E**) Gene ontology analysis of the top 10 biological processes in IEC4.1 IFN-γ-stimulated samples compared to non-treated samples. (**F**) Gene ontology analysis of the top 10 biological processes in U90926KO IFN-γ-stimulated samples compared to U90926 non-treated control. (**G**) Gene expression of known IFN-γ stimulated genes in IEC4.1 IFN-γ-stimulated samples and U90926KO IFN-γ-stimulated ones normalized to non-treated control. *P < 0.05 vs untreated IEC4.1 control cells.

IFN-γ from immune cells residing within the epithelium is essential to the cell-intrinsic defense against *Cryptosporidium* infection. Our previous studies demonstrated the host lncRNA, U90926, is upregulated during *C. parvum* infection (34), and knocking out of this lncRNA resulted in a decreased infection burden, though the underlying mechanism is unknown. Considering the important role IFN-γ plays in the defense against *C. parvum*, we speculate that U90926 may be able to modulate IFN-γ-stimulated cell-intrinsic defense. To determine if U90926 is targeting ISGs, we treated U90926 CRISPR/Cas9 knockout IEC4.1 cells (U90926KO) (Fig. S1A) with IFN-γ and performed RNA-seq analysis to compare gene expression profiles with control cells (IEC4.1). IEC4.1 cells are transformed but non-tumorigenic murine intestinal epithelial cells (34). As expected and consistent with results from previous studies (35), treatment with IFN-γ (1 ng/mL for 4 h) resulted in distinct gene expression profile changes, with known ISGs significantly upregulated in IEC4.1 cells (Fig. 1A). Significant gene expression changes were detected in U90926KO cells in response to IFN-γ stimulation compared to non-treated U90926KO samples (Fig. 1B). Interestingly, many genes were differentially expressed in U90926KO cells treated with IFN-γ compared to the IEC4.1 control cells stimulated with IFN-γ (Fig. 1C). We found that among those genes significantly upregulated in IEC4.1 cells stimulated with IFN-γ, 29% showed no change in U90926KO cells; 41% of upregulated genes were downregulated; and 31% of genes were further upregulated in the absence of U90926 with IFN-γ treatment (Fig. 1D). Gene set enrichment analysis of the gene expression profile of IEC4.1 cells treated with IFN-γ revealed various biological pathways associated with IFN-γ stimulation, including response to IFN-γ, defense response to virus, and regulation of the innate immune response (Fig. 1E). U90926KO cells stimulated with IFN-γ also revealed similar biological pathways; however, the gene count and the enrichment score differed between the two cell types (Fig. 1F). Therefore, U90926 may be targeting genes specific to the innate immune response. To expand upon this, we used unsupervised hierarchical clustering analysis, which generated a heatmap of known IFN-γ-stimulated genes to compare U90926KO cells to control cells following IFN-γ stimulation to determine if U90926 is targeting these genes specifically. Shown in Fig. S1, some IFN-γ-stimulated genes were significantly upregulated in U90926 knockout cells, including *Irgm2* and *Igtp*; some genes were significantly downregulated, including Gbp7, and others showed no significant difference. Interestingly, genes within the same response pathway or gene family did not exhibit similar changes in their expression when U90926 was knocked out. This is evident when we graph the gene expression values of U90926KO stimulated with IFN-γ and control IEC4.1 cells (Fig. 1G).

### U90926 modulates IFN-γ-stimulated gene expression in a gene-specific manner

**Fig 2:**
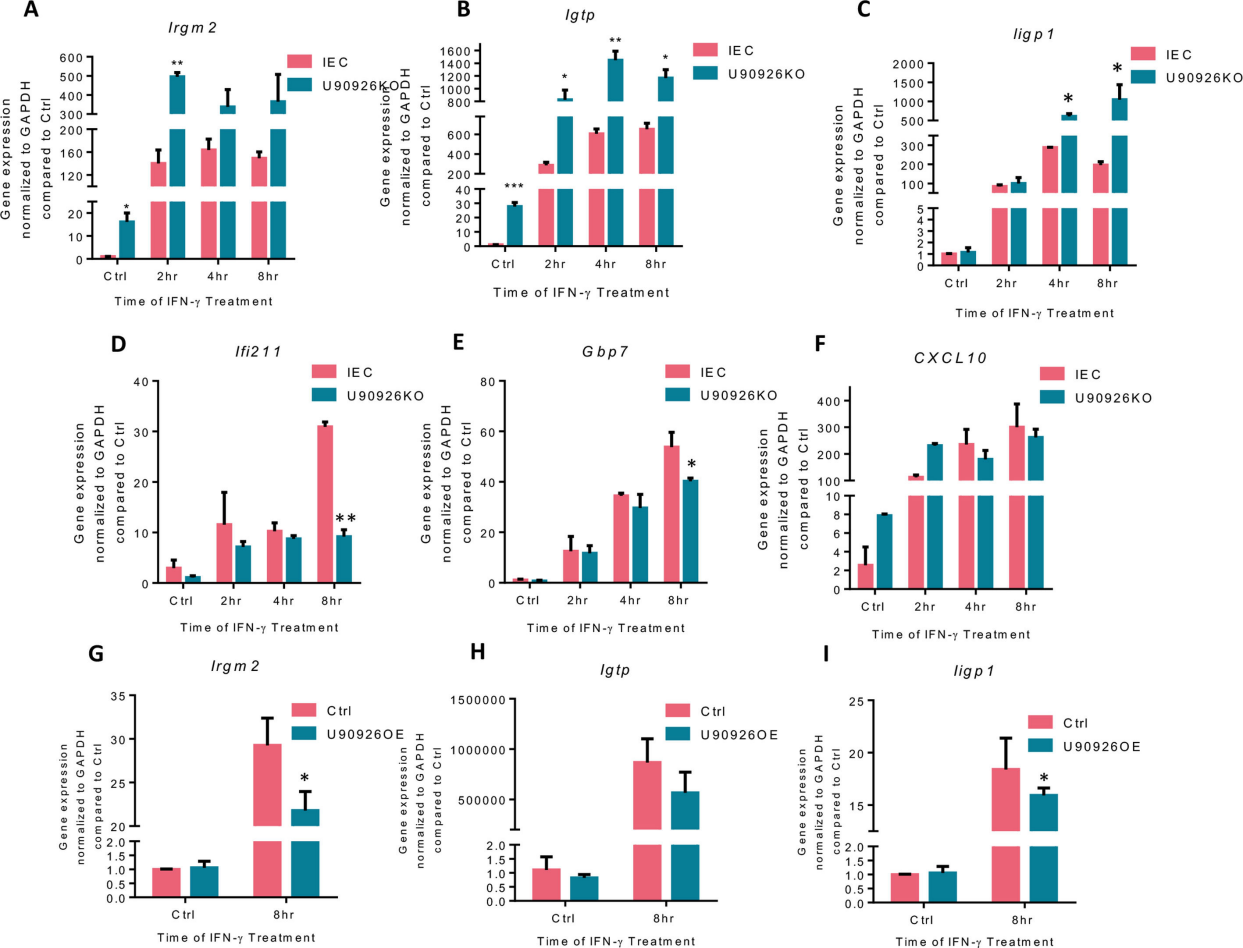
U90926 knockout alters the IFN-γ-stimulated gene expression in a gene-specific manner in intestinal epithelial cells. (**A–F**) Altered expression of IFN-γ-stimulated genes in U90926 CRISPR/Cas9 knockout cells treated with IFN-γ and compared to IEC4.1 control cells treated with IFN-γ. U90926KO and IEC4.1 cells were treated with IFN-γ for 2–8 h, and *Irgm2* (**A**), *Igtp* (**B**), *Iigp1* (**C**), *Ifi211* (**D**), *Gbp7* (**E**), and *Cxcl10* (**F**) expressions were measured via qRT-PCR. (**G–I**) ISG expression in IEC4.1 cells overexpressing U90926, followed by IFN-γ stimulation. IEC cells were transfected with a plasmid expressing U90926 RNA (U90926OE), followed by IFN-γ stimulation. Gene expression was measured via qRT-PCR for *Irgm2* (**G**), *Igtp* (**H**), and *Iigp1* (**I**). Data represent means ± SEM from three independent experiments, *P < 0.05, **P < 0.001 vs IEC4.1 untreated control.

IFN-γ produced by immune cells binds to the IFNAR1/2 receptor on intestinal epithelial cells, subsequently activating the JAK/STAT pathway. Signaling through this pathway can modulate the transcription of over 2,000 ISGs (35). To further confirm that U90926 is targeting the genes activated in this pathway in a gene-specific manner and not via the entire IFN-γ signal pathway, we employed qRT-PCR to measure the gene expression of known IFN-γ-stimulated genes in U90926KO and IEC4.1 cells overexpressing U90926. U90926KO cells or IEC4.1 control cells were treated with IFN-γ and collected at different time points (1 ng/mL for 2, 4, and 8 h); RNA was purified; and qRT-PCR was performed using primers specific to selected ISGs (Table S1). Consistent with our above RNA-Seq data (Fig. 1G), U90926KO cells treated with IFN-γ had increased expression of *Irgm2*, *Igtp* (also known as *Irgm3*), and *Iigp1* compared to IEC4.1 cells (Fig. 2A through C). In contrast, U90926KO cells treated with IFN-γ resulted in decreased expression levels of *Ifi211* and *Gbp7* and no change in the *Cxcl10* expression, all of which are activated via the JAK/STAT pathway (Fig. 2D through F). Additionally, we performed a U90926 overexpression experiment in which IEC4.1 cells were transfected with a plasmid expressing U90926 RNA (U90926OE) (Fig. S2), followed by IFN-γ treatment. qRT-PCR was then performed to measure the ISG expression. Complementary to our previous results, overexpressing U90926 in IEC4.1 cells resulted in decreased expression levels of *Irgm2*, *Igtp*, and *Iigp1* (Fig. 2G through I). Taken together, our data suggest that U90926 may be altering the IFN-γ-stimulated gene expression in a gene-specific manner and not by targeting the entire IFN-γ pathway or all IFN-γ-controlled genes.

### U90926 is recruited to the promoter regions of IFN-γ-stimulated genes to regulate transcription

**Fig 3:**
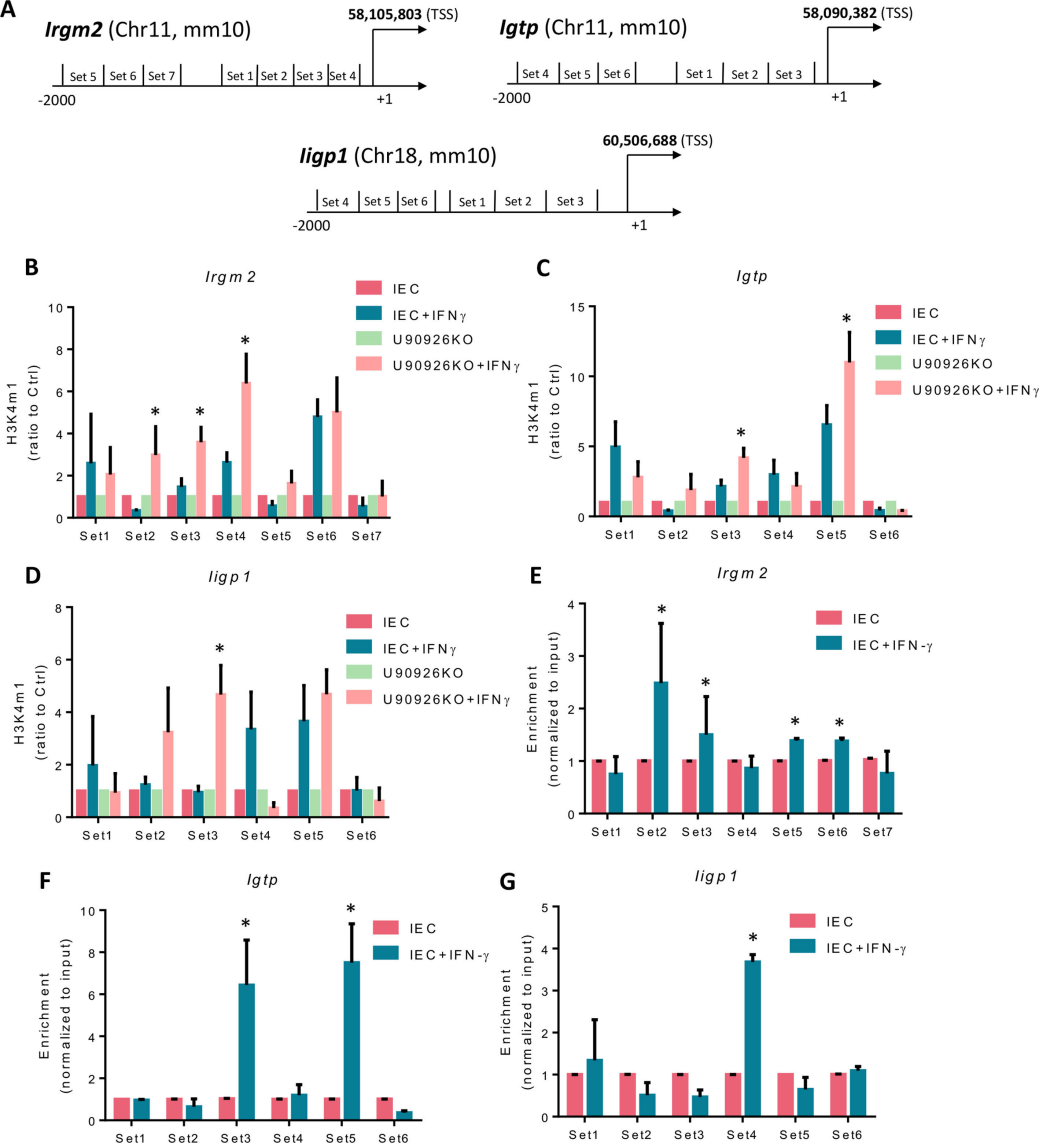
U90926 binds to the promoter regions of several IFN-γ stimulated genes to regulate their transcription. (**A**) Diagram of primer sets covering the *Irgm2*, *Igtp*, and *Iigp1* promoter regions. (**B**) Impact of U90926 on the enrichment of the activation marker H3K4m1 associated with *Irgm2*, (**C**) *Igtp*, and (**D**) *Iigp1* gene loci in CRISPR/Cas9 U90926 knockout cells, followed by IFN-γ stimulation. ChIP analysis was performed using the primer sets designed. (**E–G**) U90926 recruitment to the (**E**) *Irgm2*, (**F**) *Igtp*, and (**G**) *Iigp1* gene loci. IEC4.1 cells were treated with IFN-γ for 4 h, followed by ChIRP analysis using a pool of probes specific to U90926 and the PCR primer sets as designed. Data represent means ± SEM from three independent experiments, *P < 0.05 vs IEC4.1 untreated control.

To uncover the underlying mechanism of the gene-specific U90926 regulation of IFN-γ-stimulated genes, we sought to examine the impact U90926 might have on the transcriptional repression of *Irgm2*, *Igtp*, and *Iigp1* genes during IFN-γ stimulation. Utilizing the chromatin immunoprecipitation assay (ChIP), the histone modifications, such as H3K4m1 usually associated with gene transactivation, can be examined in the presence and absence of U90926 following the IFN-γ stimulation. IEC4.1 and U90926KO cells were treated with IFN-γ, and the enrichment of H3K4m1 was measured using qPCR primers designed to cover the various regions of regulatory promoters within the three gene loci (Fig. 3A). U90926KO cells stimulated with IFN-γ had significantly higher H3K4m1 levels in the *Irgm2* gene locus compared to IEC4.1 control cells stimulated with IFN-γ, specifically at locations 2, 3, and 4 (Fig. 3B). The *Igtp* gene locus also displayed significantly increased levels of H3K4m1 at locations 3 and 5 (Fig. 3C) when U90926 was knocked out. Additionally, there was significantly more H3K4m1 enrichment at locations 2 and 3 in the *Iigp1* gene locus (Fig. 3D). Furthermore, we examined how the transcription of *Irgm2*, *Igtp*, and *Iigp1* genes is selectively regulated by U90926 and whether this lncRNA is recruited directly to these gene loci. We utilized a chromatin isolation by RNA purification assay (ChIRP) to measure the occupancy of U90926 at the gene loci. A pool of biotinylated tiling oligonucleotide probes with an affinity for the U90926 sequence was used to precipitate the chromatin fragments via glutaraldehyde cross-linking and chromatin isolation. The same qPCR primers used for the ChIP assay were employed to identify the DNA sequences of the precipitated chromatin fragments. We detected an increase in the presence of U90926 in the gene loci of all three genes in IEC4.1 cells treated with IFN-γ. Irgm2 displayed increased levels of U90926 at locations 2, 3, 5, and 6 (Fig. 3E); Igtp showed significantly increased levels at locations 3 and 4 (Fig. 3F); and Iigp1 displayed a significant increase in U90926 at location 4 of the gene locus (Fig. 3G). Taken together, these data indicate that U90926 may be altering IFN-γ-stimulated gene transactivation via altered H3K4m1 enrichment, and U90926 is recruited directly to the gene loci of *Irgm2*, *Igtp*, and *Ilgp1*.

### U90926 interacts with the Ehmt2 complex to aid in enrichment in the promoter region of IFN-γ stimulated genes to alter gene transactivation

**Fig 4:**
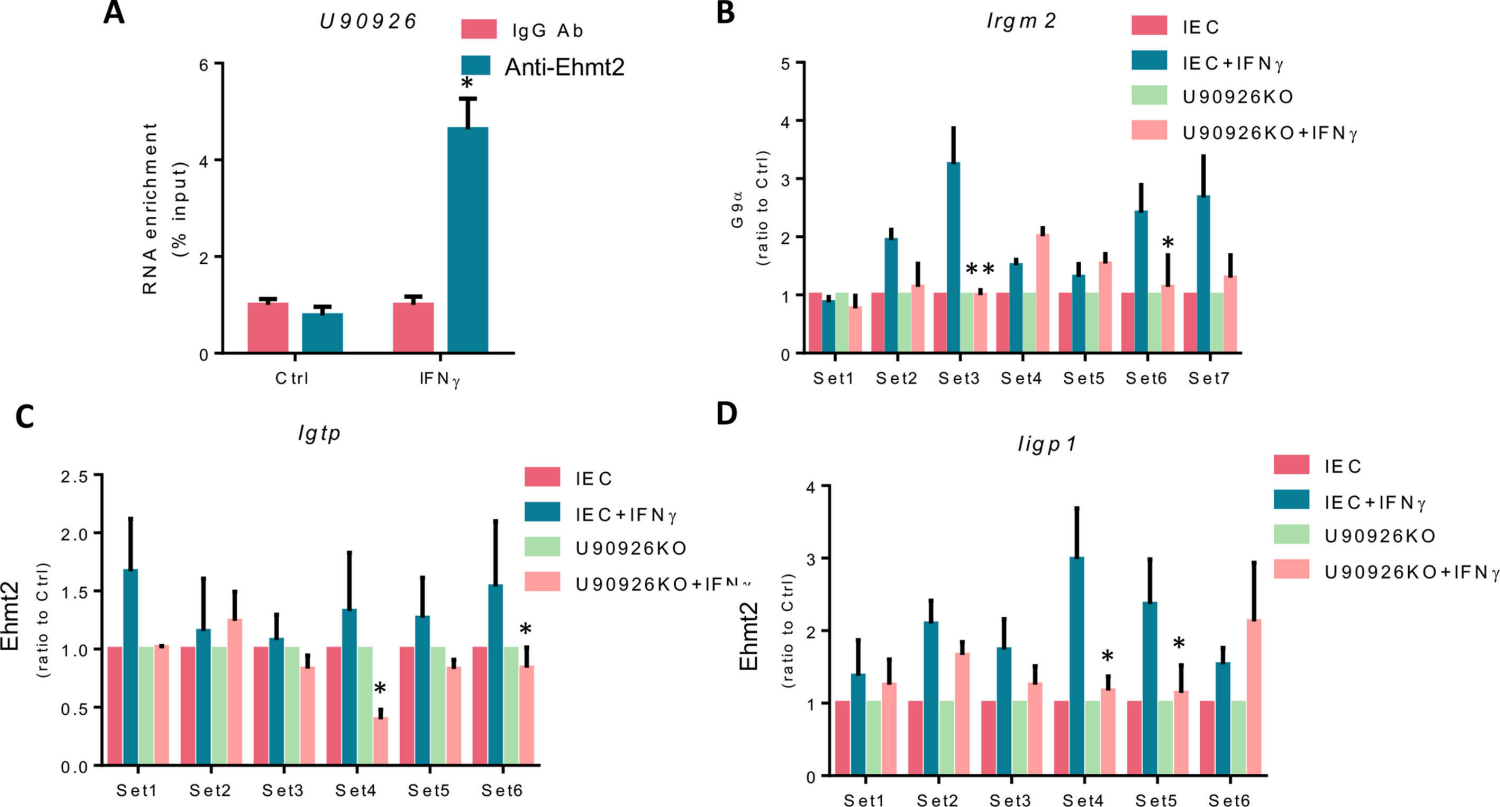
U90926 interacts with Ehmt2 and alters Ehmt2 enrichment in the promoter region of IFN-γ-stimulated genes. (**A**) RIP assay for U90926 from IEC cells treated with IFN-γ was performed using an Ehmt2 antibody, and isotype IgG was used as a negative control. (**B**) ChIP analysis of Ehmt2 in the promoter region of IFN-γ-stimulated genes *Irgm2* (**B**), *Igtp* (**C**), and *Iigp1* (**D**) in U90926KO cells treated with IFN-γ. Data represent means ± SEM from three independent experiments, *P < 0.05 vs IEC4.1 untreated control.

**Fig 5:**
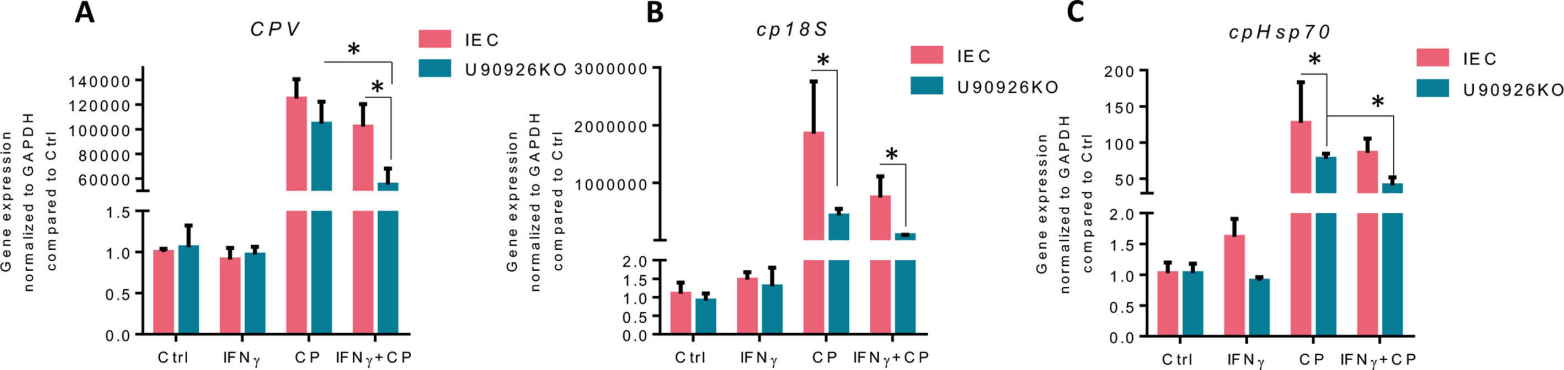
U90926 restricts the IFN-γ-mediated intestinal epithelia cell-intrinsic anti-*Cryptosporidium* defense. (**A–C**) Knockout of U90926 results in decreased *Cryptosporidium* infection. U90926 CRISPR/Cas9 knockout cells were treated with IFN-γ 16 h before infection with *C. parvum*. After 8 h of infection, infection burden was measured via qRT-PCR with primers specific for (**A**) CPV, (**B**) cp18S, and (**C**) cpHsp70 and compared to the non-IFN-γ-treated control. Data represent means ± SEM from three independent experiments, *P < 0.05 vs IEC4.1 untreated control.

**Fig 6:**
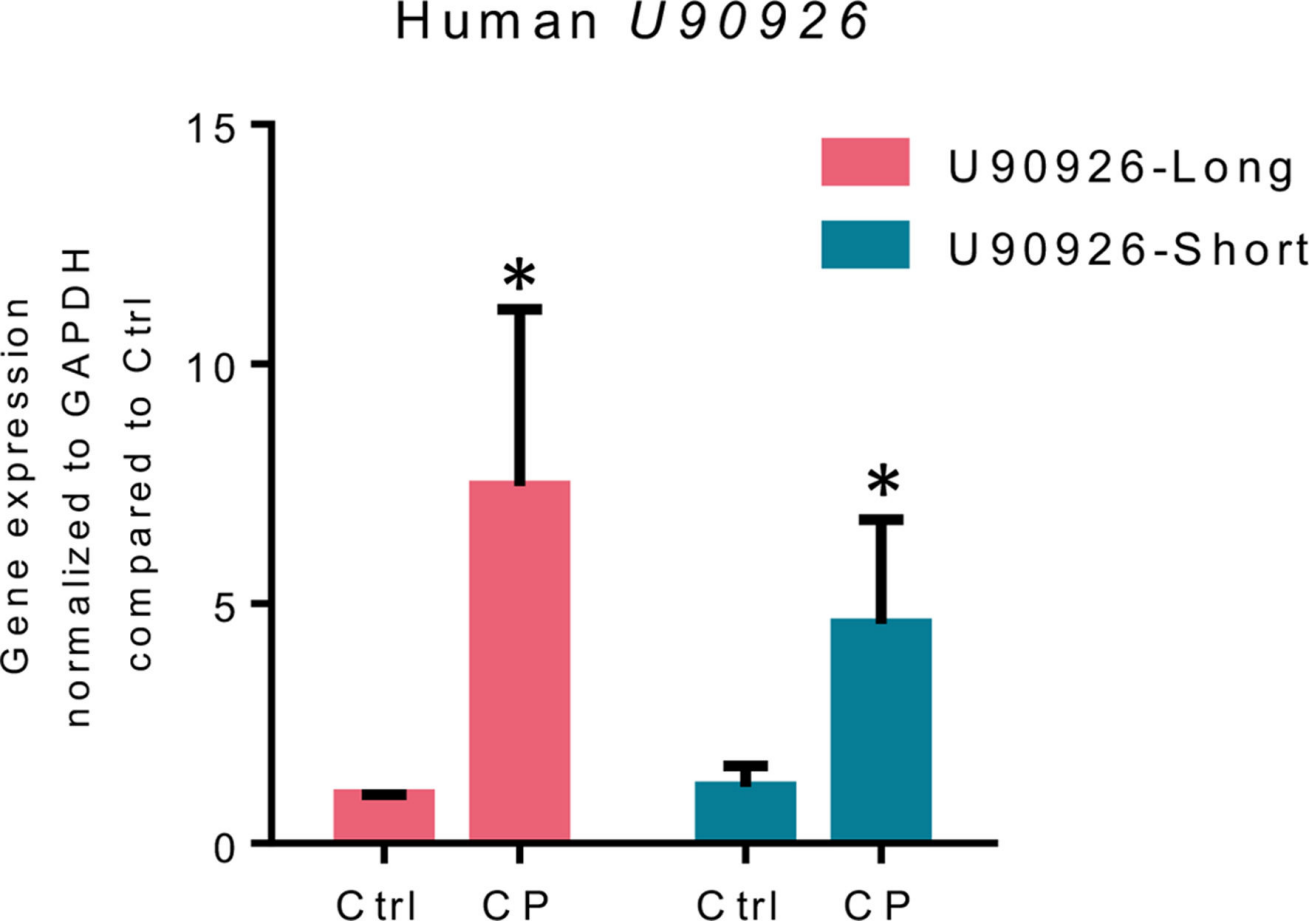
U90926 is upregulated in human intestinal epithelial cells during *C. parvum* infection. Human intestinal adenocarcinoma cells (HCT-8) were infected with *C. parvum*, and the expression levels of the short and long transcripts were measured via qRT-PCR. Data represent means ± SEM from three independent experiments, *P < 0.05 vs HCT-8 uninfected control.

The euchromatic histone lysine methyltransferase 2 (Ehmt2), a histone methyltransferase for H3 methylation, mediates gene trans-suppression in many cell types (36). Current research suggests that Ehmt2 associates with the H3K4 demethylase Jarid1a at the promoter region of genes repressing their transcription (37). We wondered if Ehmt2 is involved in the U90926-altered H3K4m1 enrichment of IFN-γ stimulated genes. Utilizing a cross-linking RNA immunoprecipitation (RIP) assay, we found a significant amount of U90926 in the anti-Ehmt2 immunoprecipitates from IFN-γ Ehmt2-stimulated IEC4.1 cells compared to the non-treated control (Fig. 4A). To determine if U90926 alters Ehmt2 enrichment at the promoter region of IFN-γ stimulated genes, we performed a ChIP assay using an Ehmt2 antibody and the same qPCR primers from Fig. 3, covering the various regions of regulatory promoters in *Irgm2*, *Igtp*, and *Iigp1* in U90926KO cells stimulated with IFN-γ. We found significantly reduced levels of Ehmt2 in the promoter region of all three genes when U90926 was knocked out compared to control IEC4.1 cells. These were specifically at locations 3 and 6 in the *Irgm2* gene locus (Fig. 4B), 4 and 6 in the *Igtp* gene locus (Fig. 4C), and 4 and 5 in the *Iigp1* gene locus (Fig. 4C). These data indicate that U90926 interacts with Ehmt2 and is involved in Ehmt2 enrichment to the promoter region of the IFN-γ-stimulated genes tested in IEC4.1 cells stimulated with IFN-γ.

### U90926 restricts IFN-γ-mediated intestinal epithelial cell-intrinsic anti-*Cryptosporidium* defense

We previously showed that U90926 acts in a pro-parasitic manner in intestinal epithelial cells, and inhibiting this lncRNA resulted in a decrease in the *C. parvum* infection burden (34). To determine if U90926’s impact on the IFN-γ-stimulated gene transcription influences the IFN-γ-mediated intestinal epithelial cell-intrinsic defense against *C. parvum*, we measured the infection burden in U90926KO cells pre-treated with IFN-γ, followed by *C. parvum* infection. U90926KO or IEC4.1 cells were treated with IFN-γ 16 h before infection with *C. parvum*. After 8 h of infection, the samples were collected, and qRT-PCR was performed to measure *C. parvum* 18s (cp18s), *Cryptosporidium* parvum virus (CPV), and Hsp70 (cpHsp70) levels, reflecting the parasite burden. Consistent with previous studies (38, 39), we detected a decrease in the infection burden in IEC4.1 cells pre-treated with IFN-γ. U90926KO cells demonstrated significantly lower levels of infection burden compared to IEC4.1 cells, which were previously shown (34). Pre-treatment with IFN-γ in U90926KO cells decreased the *C. parvum* infection burden even further compared to IEC4.1 cells with IFN-γ pre-treatment and U90926KO cells without pre-treatment (Fig. 5A through C). Effects of U90926KO on the IFN-γ-mediated anti-*Cryptosporidium* defense were further confirmed by immunofluorescent staining (Fig. S3). These data suggest that U90926 is targeting the IFN-γ-stimulated gene expression to inhibit the IFN-γ-mediated epithelial cell-intrinsic anti-*Cryptosporidium* defense.

### U90926 is upregulated in human intestinal epithelial cells during *C. parvum* infection

Thus far, we have shown that U90926 is upregulated during *C. parvum* infection and is targeting ISGs in a gene-specific manner to inhibit the cell-intrinsic antiparasitic defense in mice. However, U90926’s role in the human antiparasitic defense is unknown. The human transcript of U90926 was identified in the vitreous fluid of patients with acute retinal necrosis caused by HSV-1 infection, and it encodes short and long transcript variants and appears to be evolutionarily conserved between mice and humans (40). Using the primers from this report (41), we infected human intestinal adenocarcinoma (HCT-8) cells with *C. parvum* and measured the expression of the short and long variants of human U90926 via qRT-PCR. We found that both the long and short variants of U90926 were significantly upregulated in HCT-8 cells following *C. parvum* infection (Fig. 6). These data suggest that U90926 upregulation during *C. parvum* infection is a conserved mechanism between mice and humans.

## DISCUSSION

The intestinal epithelial cells are the first line of defense against intracellular pathogens and play a key role in activating the intestinal immune response. Within the intestinal epithelial cells are layered innate immune signaling networks that operate to detect microbial pathogens and stimulate downstream pathogen elimination pathways, including the production of antimicrobial proteins, specialized degradative compartments, and programmed host cell death. A main component of the anti-*Cryptosporidium* defense is IFN-γ, a cytokine released during infection that is responsible for the transcription of over 2,000 genes with anti-pathogenic consequences (35). Emerging evidence highlights the role of long non-coding RNAs in the regulation of gastrointestinal defense against microbial challenge. In this study, we sought to determine the role that U90926, a host lncRNA previously shown to aid *Cryptosporidium* during infection, may be playing in regulating the IFN-γ-mediated anti-*Cryptosporidium* defense. Our findings indicate that U90926 is targeting the transcription of some IFN-γ ISGs by altering histone modifications associated with gene transactivation. U90926 appears to be interacting with Ehmt2, a euchromatic histone lysine methyltransferase, in the promoter region of the IFN-γ-ISGs to suppress transcription and ultimately inhibit the anti-*Cryptosporidium* defense.

Upon infection with *Cryptosporidium*, a strong cell-mediated response is induced in which IFN-γ plays a leading role in driving the cellular immune response and effector mechanisms against the parasite. Sources of IFN-γ include NK cells, macrophages, T cells, and innate lymphocyte cells (ILC1s) that infiltrate the site of infection. IFN-γ binds to the heterodimeric receptor of IFNGR1 and IFNGR2 expressed on intestinal epithelial cells to activate the JAK/STAT signaling pathway. Signaling through this pathway results in the expression of various ISGs that drive pathogen elimination through mechanisms like nutrient deprivation and the production of potent defense molecules like reactive oxygen and nitrogen species, guanylate-binding proteins (GBPs), and immunity-related GTPases (IRGs) (41). Among these IRGs are the genes *Irgm2*, *Igtp* (also known as *Irgm3*), and *ligp1*. The exact mechanisms of anti-*Cryptosporidium* defense by these genes are not fully understood, but they appear to have essential and pathogen-specific roles in resistance to infection (16). One study found that the expression levels of *Irgm2* and *Igtp* increase during *Cryptosporidium* infection (42), and mice lacking *Irgm2* and *Igtp* have been shown to be more susceptible to infection compared to noninfected control mice (41). During *Toxoplasma gondii* infection, IRGs have been reported to localize at the pathogen-containing vacuole and disrupt the membrane (43). Further experimentation is required to understand the exact mechanisms of defense; however, it would be beneficial for the parasite to evolve strategies to dysregulate the IFN-γ-mediated response. We show here that U90926 is targeting some IFN-γ ISGs in a gene-specific manner to repress their expression during *C. parvum* infection, suggesting a potential strategy used by *C. parvum* to evade the host immune response.

LncRNAs can regulate gene expression through many different mechanisms involving specific interactions with DNA, RNA, and proteins. They can act as signals, scaffolds, decoys, or guides to alter histone modifications, regulate transcription, alter mRNA stability, or regulate translation (44). We show here that U90926 is regulating IFN-γ ISG expression by altering the enrichment of active histone modifications in the promoter regions of *Irgm2*, *Igtp*, and *Iigp1*. U90926 is recruited to the promoter regions of these genes and directly interacts with Ehmt2, a euchromatic methyltransferase. In the absence of U90926, there was significantly less Ehmt2 in the promoter region of *Irgm2*, *Igtp*, and *Iigp1* and significantly more H3K4m1 methylation. Ehmt2 can act as both a repressor or an activator of transcription depending on the interaction with other transcriptional co-activators or repressors (45). A recent study found Ehmt2 binds directly to Jarid1a, a H3K4 demethylase, to remove active histone marks H3K4m1 and repress gene transcription (37). Our data demonstrate that as a mechanism of action, U90926 and Ehmt2 alter active histone marks in the promoter region of IFN-γ ISGs to suppress their expression during *C. parvum* infection. More research will need to be done to determine if Jarid1a is involved.

 The success of the parasite ultimately relies on its ability to utilize host machinery and resources while evading the host immune response. This evolutionary pressure drives the host and the parasite into a constant co-evolutionary arms race in which the parasite evolves novel strategies to escape the immune response, and the immune system simultaneously evolves strategies to kill the parasite. We demonstrate here a novel strategy in which *C. parvum* hijacks a host lncRNA to repress the IFN-γ mediated response. We show that in the absence of U90926, pre-treatment with IFN-γ significantly reduced the levels of parasite burden in intestinal epithelial cells compared to when the lncRNA was present. Taken together, these findings suggest a strategy of immune evasion by *C. parvum* through induction of a host lncRNA.

 Additionally, while most of the work shown here was performed in mouse infection models of cryptosporidiosis, we did identify the human U90926 transcript following *C. parvum* infection in human ileocecal colorectal adenocarcinoma cells. A recent study came out looking at the vitreous fluid of patients with acute retinal necrosis caused by HSV-1 infection in which they identified two potential transcripts of human U90926, long and short versions, suggesting this lncRNA is conserved between species (40). We found that both versions of the U90926 transcript were upregulated following *C. parvum* infection, suggesting that this is a conserved mechanism of evasion for *C. parvum*. More research needs to be done to determine if U90926 is regulating the human IFN-γ-mediated anti-*Cryptosporidium* defense in a similar way as in mice.

## MATERIALS AND METHODS

### Cell cultures

The mouse intestinal epithelial cell lines were graciously provided by Dr. Pingchange Yang of McMaster University in Hamilton, Canada. Cultures of IEC4.1 cells were maintained in DMEM media with 10% fetal bovine serum (FBS; Gibco; Caldbad, CA). Streptomycin at 100 µg/mL and penicillin at 100 U/mL were added to the media, and cultured cells were kept at 37°C in a 5% CO_2_ incubator. Stable IEC4.1 cells deficient in U90926 were generated through transfection with the CRISPR/Cas9 KO(h) (U90926-CRISPR/Cas9 KO) and the HDR plasmid (U90926-HDR) as previously described (46).

### *C. parvum* oocysts

*C. parvum* oocysts of the Iowa strain provided by Harley Moon at the National Animal Disease Center (Ames, IA) were purchased from a commercial source (Bunch Grass Farm, Deary, ID). Oocysts were purified using a modified ether extraction technique. The oocysts were suspended in phosphate-buffered saline (PBS) and stored at 4°C. For i*n vitro* infection experiments, the oocysts were subjected to a treatment with 1% sodium hypochlorite while being preserved on ice for 20 min. After this treatment, the oocysts were washed three times using Dulbecco’s modified Eagle medium (DMEM) culture media.

### *C. parvum* infection assay

Models of intestinal cryptosporidiosis using intestinal epithelial cell lines were employed as previously described (33, 47). Viable oocysts treated with 1% sodium hypochlorite were added to a culture medium consisting of DMEM-F-12 supplemented with 100 U/mL penicillin and 100 µg/mL streptomycin. The infection was established by combining the oocysts and host cells in a 1:1 ratio. The infected cell cultures were incubated at 37°C for 4 h to facilitate parasite attachments and invasion. Cells were thoroughly washed with DMEM-F-12 medium three times to remove any free parasites. The cells were further cultured for varying time periods based on the experimental requirement. Infection burden was quantified by quantitative real-time PCR (qRT-PCR) measuring levels of *C. parvum* 18s (*cp18s*) or Hsp70 (*cpHsp70*) gene and by immunofluorescent staining (33, 38). Immunofluorescent staining of *C. parvum* was carried out using the rabbit antiserum against *C. parvum* membrane proteins as previously described (38).

### U90926 overexpression

The sequence of U90926 was amplified through PCR and cloned into the pTarget vector according to the manufacturer’s protocol. U90926-pTarget vector was transfected into cells with Lipofectamine 2000 (Invitrogen). Control cells were transfected with an empty pTarget vector. qRT-PCR was used to determine alterations in gene expression.

### RNA isolation

Total RNA from the cells was extracted using the TRIzol reagent from Invitrogen as per the manufacturer’s instructions. The concentration and the quality of the RNA were assessed using a spectrophotometer from Beckman in Brea, CA. For subsequent steps, 500 ng of the RNA sample was used as a template for cDNA synthesis performed using the M-MLV Reverse Transcriptase Kit from Invitrogen. The reaction was carried out in a total volume of 20 µL.

### qRT-PCR

qRT-PCR was performed using Invitrogen SYBR GreenER qPCR SuperMix Universal from Thermo Fisher Scientific and on the Bio-Rad CFX96 Touch Real-Time PCR Detection System. The expression levels of the RNA samples were determined using the threshold cycle (ΔΔCT) method and normalized to the housekeeping gene glyceraldehyde-3-phosphate dehydrogenase (GAPDH). The primer sequences are shown in Table S1.

### RNA sequencing and bioinformatics analysis

RNA-Seq was performed in biological triplicates in IEC4.1 cells and CRISPR/Cas9 U90926 knockout cells treated with or without IFN-γ (1 ng/mL for 4 h). Total RNA molecules were isolated using Trizol reagent. The BGI Americas Corporation in Cambridge, MA performed transcriptome sequencing and data processing using DNBSEQ sequencing technology platforms. To assay the RNA quality, the 2100 Bioanalyzer from Agilent Technologies was employed. The complete RNA was broken down into smaller fragments, and specific mRNA molecules were concentrated using magnetic beads with oligo (dT) sequences. Subsequently, complementary DNA was synthesized from the enriched mRNA. This cDNA then underwent purification and was amplified using PCR. The sequenced library products were processed on the DNBSEQ-500 platform with paired-end reads of 100 base pairs. To ensure data quality, subpar reads were excluded using the internal software, called SOAPnuke on the DNBSEQ-500 platform. The resultant high-quality reads were stored in the FASTQ format. Reads were aligned to the Mus_musculus genome (GCF_000001635.26_GRCm38.p6) from the National Center for Biotechnology Information data using two different software tools (i.e., HISAT2 and Bowtie2). The alignment was guided by parameters tailored to the specifics of the process. Raw RNA-seq data were further refined by filtering out unreliable sites through the GATK program. This step targeted the removal of low-quality reads from each step. Finally, the gene read counts were normalized to RPKM, which represents the expression level of genes in terms of reads per kilobase of transcript per million mapped reads. This normalization allowed for a meaningful comparison of relative gene expression levels across samples.

Differential gene expression analyses were conducted using DESeq2. Differences in gene expression were quantified using logarithmically transformed fold change values. These values were represented as either log2FC or log10FC (log base 10) and computed as the logarithmic difference between gene expression values under distinct treatment conditions (log2FC = log2[B] − log2[A] or log10FC = log10[B] – log10[A], where A and B represent the gene expression values). To further analyze the data, the gene set enrichment analysis (GSEA) was conducted using the “clusterProfiler” R package. This analysis was carried out in a pre-ranked manner, where all genes were ranked based on their log2FC values derived from the differential expression analysis. The weighted Kolmogorov-Smirnov test was applied, and *P*–values were adjusted using the Benjamini-Hochberg method. The findings from GSEA were depicted visually using the “ggplot2” R package. Additionally, the volcano plots were generated with the “ggplot2” package utilizing log2FC and the adjusted *P*-values. A heatmap was generated using the “ComplexHeatmap” R package to visually summarize the gene expression patterns across different conditions. Additionally, the expression of the *z*-score was computed based on relative gene expression levels to aid in standardizing and comparing gene expression values across diverse samples, facilitating a more meaningful interpretation of expression patterns.

### Chromatin immunoprecipitation (ChIP) assay

The detailed protocol has been previously described elsewhere (48, 49). Briefly, cells were treated with 1% formaldehyde for 10 min to cross-link the proteins to the DNA, preserving the protein-DNA interactions at the time of fixation. The fixed cells were collected in 1× ice-cold phosphate-buffered saline (PBS) and resuspended in a sodium dodecyl sulfate (SDS) lysis buffer. The genomic DNA was then fragmented into smaller pieces through sonication, resulting in fragments ranging from 200 to 500 base pairs in length. One percent of the cell extract was kept aside as input. The cell extracts containing the sheared DNA were incubated overnight at 4°C with anti-Stat1 (or anti-H3k4m1) and added protein G-agarose beads to precipitate the antibody-protein-DNA complexes. The immunoprecipitates (antibody-protein-DNA complexes bound to the beads) were washed sequentially to remove any non-specific interactions. The washing steps involve low- and high-salt buffers, LiCl buffer, and Tris EDTA buffer. The DNA-protein complexes were eluted from the beads afterwards, separating the DNA from the proteins. The proteins in the eluted solution were then digested at 45°C for 1 h using protease K. To detect and quantify the DNA, qRT-PCR analysis was employed, enabling the quantification of protein binding levels at particular regions of the genome; primers are listed in Table S1.

### Chromatin isolation by RNA purification (ChIRP) assay

Chromatin isolation by RNA purification (ChIRP) analysis was conducted as described in a previous study (34). To summarize, chromatin was isolated after cross-linking the cells with glutaraldehyde. A set of tiling oligonucleotide probes was designed to specifically bind to the U90926 sequence (sequences listed in Table S1). Verification of the DNA sequences in the chromatin immunoprecipitates was performed using qRT-PCR. The same primers used in the ChIP assay covering the promoter region of the genes of interest were used, and a collection of scrambled oligonucleotide probes targeting LacZ (found in Table S1) was used as the control.

### RNA immunoprecipitation assay

The formaldehyde crosslinking RIP was performed as described (50). Briefly, lysates were precleaned with PBS-washed Magna ChIP protein A + G magnetic beads (Millipore). The precleaned lysates were then diluted with whole cell extraction buffer, mixed with specific antibody-coated beads, and then incubated with rotation at 4°C for 4 h. Samples were then washed four times with whole cell extraction buffer containing protease and RNase inhibitors. The collected immunoprecipitated RNP complexes and input were digested in an RNA PK buffer pH 7.0 (100 mM NaCl, 10 mM Tris-HCl pH 7.0, 1 mM EDTA 0.5% SDS) with protease K and incubated at 50°C for 45 min with end-to-end shaking at 400 rpm. Formaldehyde cross-links were reversed by incubation at 65°C with rotation for 4 h. RNA was extracted from these samples using Trizol according to the manufacturer’s protocol (Invitrogen) and treated with a DNA-free DNase Treatment and Removal I Kit according to the manufacturer’s protocol (Ambion). The presence of RNA was measured via qRT-PCR. Gene-specific PCR primer pairs are listed in Table S1. The following antibodies were used for RIP analysis: anti-G9α (Cell Signaling Technology) and mouse IgG (Santa Cruz).

### Statistical analysis

Data are given as mean ± SEM from at least three independent experiments or biological replicates. The two-tailed unpaired Student’s *t*-test was used for comparison between two groups, and one-way analysis of variance was used for comparison among three or more groups. *P*-values < 0.05 were considered statistically significant.

## Data Availability

The RNA-seq data used in this study are deposited in the Gene Expression Omnibus (GEO) database repository under accession number GSE245533.

## SUPPLEMENTAL MATERIAL

Supplemental figures iai.00328-25-s0001.pdf Fig. S1 to S3.

Table S1 iai.00328-25-s0002.xlsx List of primers used for the qPCR, ChIP, and ChIRP.

ASM does not own the copyrights to Supplemental Material that may be linked to, or accessed through, an article. The authors have granted ASM a non-exclusive, world-wide license to publish the Supplemental Material files. Please contact the corresponding author directly for reuse.
