# Supplementary material for: Long non-coding RNA U90926 modulates IFN-γ-stimulated gene transcription and cell-intrinsic anti-Cryptosporidium defense in intestinal epithelial cells
Source: Infect Immun. 2025 Sep 22;93(10):e00328-25. doi: 10.1128/iai.00328-25 (PMC12519777; doi:10.1128/iai.00328-25)
Supplement: Supplemental figures — Fig. S1 to S3. [file iai.00328-25-s0001.pdf]

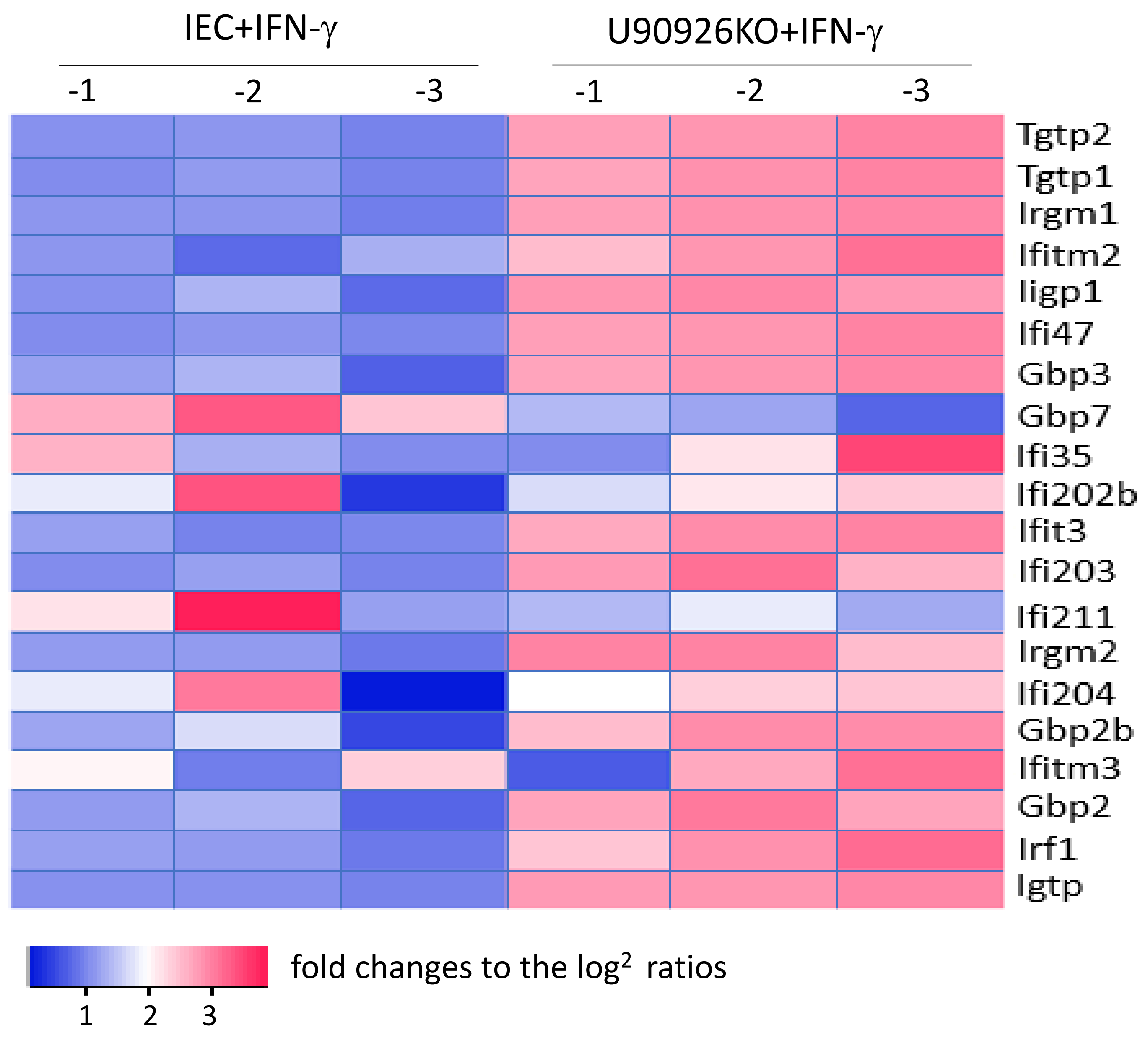


**Supplemental Figure 1.** Top significantly altered genes in IFN-γ-stimulated U90926KO-IEC4.1 cells compared to IFN-γ stimulated IEC4.1 cells. Cells were treated with IFN-γ (1 ng/ml for 4h) followed by RNA-seq analysis. Heatmap representing known IFN-γ-stimulated genes in cells following IFN-γ treatment, presented as fold changes to the mean value of the $log^2$ ratios in the IEC4.1 non-treated control (n=3).

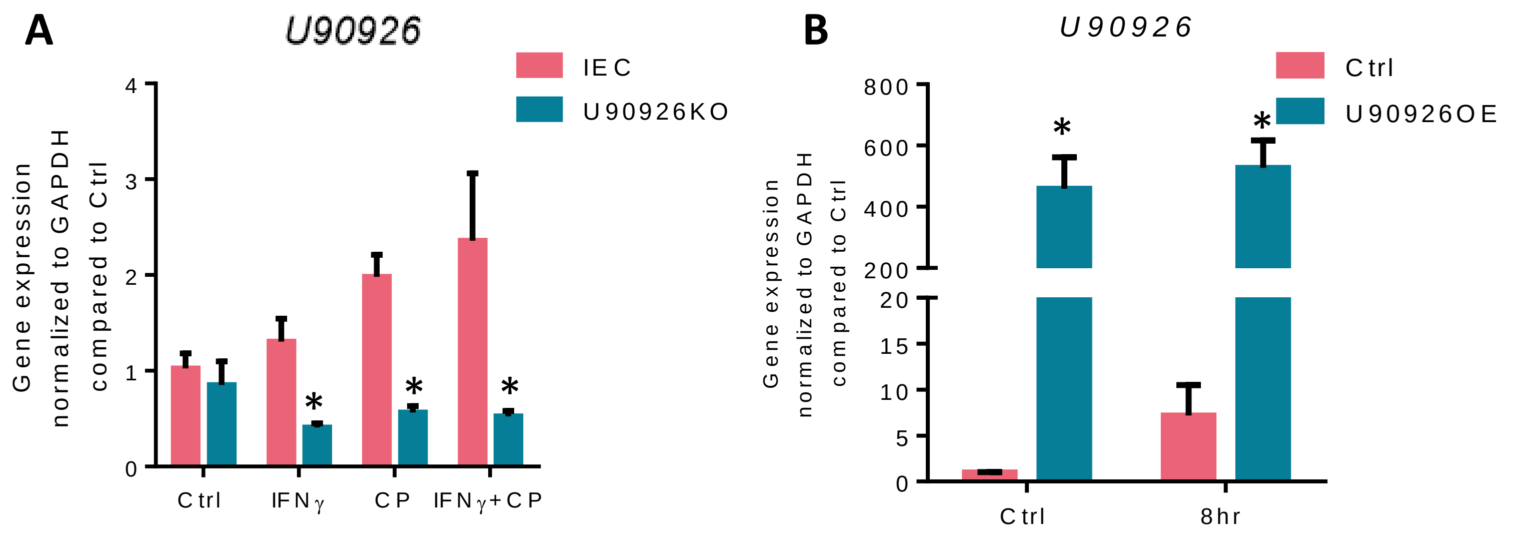


**Supplemental Figure 2.** U90926 expression in CRISPR/Cas9 knockout and siRNA overexpression cells. (A) U90926 expression in U90926 CRISPR/Cas9 knockout cells. U90926 CRISPR/Cas9 knockout cells were generated and U90926 expression was measured after *C. parvum* (CP) infection or IFN-γ stimulation via qRT-PCR and compared to wildtype IEC cells. (B) U90926 expression in IEC cells transfected with a plasmid carrying U90926 RNA. IEC cells were transfected with a plasmid carrying U90926 RNA and U90926 expression was measured via qRT-PCR and compared to IEC cells transfected with a scrambled siRNA control. Data represents means ± SEM from three independent experiments, * $p<0.05$ vs IEC4.1 untreated control.

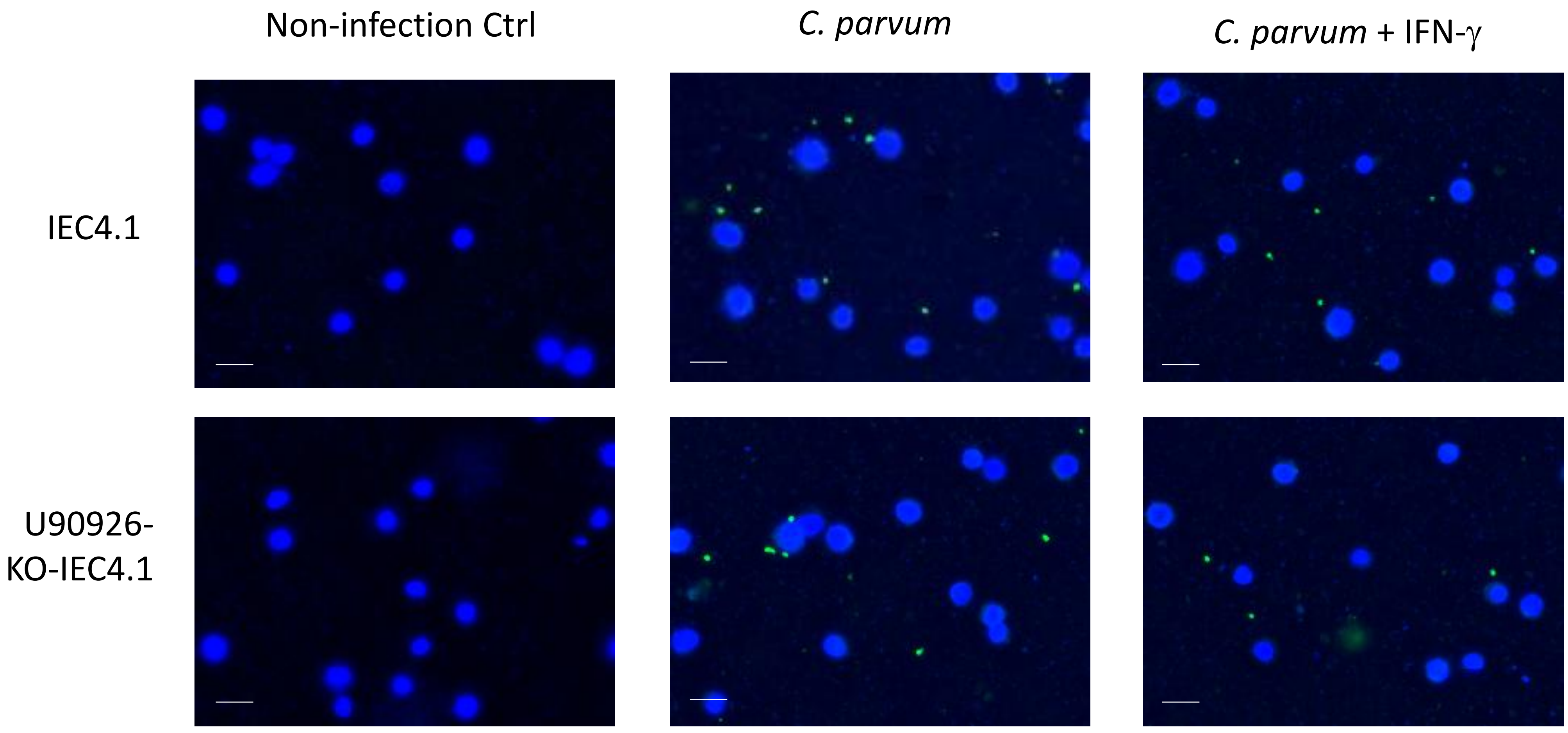


**Supplemental Figure 3.** Knockout of U90926 results in an increase of IFN-γ-mediated intestinal epithelia cell-intrinsic anti-*Cryptosporidium* defense. U90926 CRISPR/Cas9 knockout cells were with IFN-γ 16hr before infection with *C. parvum*. After 8hr of infection, infection was measured via immunofluorescence staining. Representative images show *C. parvum* parasites in green and cell nuclei labeled with DAPI (4',6-diamidino-2-phenylindole) in blue. Bar = 10 µm.
